# Peptide Vocabulary Analysis Reveals Ultra-Conservation and Homonymity in Protein Sequences

**DOI:** 10.4137/bbi.s415

**Published:** 2009-11-24

**Authors:** Derek Gatherer

**Affiliations:** MRC Virology Unit, Institute of Virology, Church Street, Glasgow G11 5JR UK

**Keywords:** peptide vocabulary, vocabulary analysis, word detection, motif, protein structure, bioinformatics, gene families, genome signature, peptide conservation, peptide homonymity

## Abstract

A new algorithm is presented for vocabulary analysis (word detection) in texts of human origin. It performs at 60%–70% overall accuracy and greater than 80% accuracy for longer words, and approximately 85% sensitivity on *Alice in Wonderland*, a considerable improvement on previous methods. When applied to protein sequences, it detects short sequences analogous to words in human texts, i.e. intolerant to changes in spelling (mutation), and relatively context-independent in their meaning (function). Some of these are homonyms of up to 7 amino acids, which can assume different structures in different proteins. Others are ultra-conserved stretches of up to 18 amino acids within proteins of less than 40% overall identity, reflecting extreme constraint or convergent evolution. Different species are found to have qualitatively different major peptide vocabularies, e.g. some are dominated by large gene families, while others are rich in simple repeats or dominated by internally repetitive proteins. This suggests the possibility of a peptide vocabulary signature, analogous to genome signatures in DNA. Homonyms may be useful in detecting convergent evolution and positive selection in protein evolution. Ultra-conserved words may be useful in identifying structures intolerant to substitution over long periods of evolutionary time.

## Introduction

First used at least as early as the beginning of the 1970s, the concept of “the language of the genes” has become a recurring explanatory tool in popular presentations of molecular genetics (Chargaff, 1971; Jones, 1993). Genomes may be compared to libraries of genetic information, with each chromosome as a book, genes as chapters, and DNA bases as the letters in which the text is written (Ridley, 1999). In principle, the linguistic analogy may be applied equally to protein sequences as to DNA, simply by increasing the alphabet from 4 to 20 letters. The prevalence, and utility, of this metaphor in undergraduate teaching and the popular science media, obscures a deeper controversy concerning its genuine applicability in research (Searls, 1993; Ji, 1999; Searls, 2002; Sakakibara, 2005). Attempts have been made to apply generative grammar structures to gene organization in bacteria (Collado-Vides, 1991, 1992, 1996), DNA-protein interaction (Bentolila, 1996; Wang et al. 2005), the problem of gene prediction (Dong and Searls, 1994; Muggleton et al. 2001), protein folding (Gimona, 2006) and RNA structure prediction (Matsui et al. 2004). These efforts in molecular biology are in the tradition of wider attempts to create formal grammars, or to use the grammatical metaphor, for other kinds of biological data (Gutfreund, 1976; Jerne, 1985; Hamilton, 1993; Wang, 2004). A related metaphor is that of genome sequence as a code to be deciphered by the molecular biologist, who thus becomes a “biomolecular cryptologist” (Konopka, 1994; Bodnar et al. 1997). Conversely, techniques developed in molecular biology are now being recycled back into cryptography (Spencer et al. 2004).

Under the terms of these general analogies, short sequences of DNA may be regarded as *words*. Often, any *k*-mer is referred to as a word (Mantegna et al. 1994; Chatzidimitriou-Dreismann et al. 1996) but here these will be designated *strings*. Where a string has some local functional significance in a sequence and consequently has been conserved throughout the evolutionary process, it may be referred to as a *motif* (Waterman, 1989; Hu et al. 2000). Identification of motifs is usually based on large-scale comparative analysis and alignment of related sequences.

Counts of DNA string frequency have been used as a means of differentiating classes of DNA sequence, such as exons, introns and promoters (Beckmann et al. 1986; Solovyev and Lawrence, 1993; Solovyev et al. 1994b, 1994a; Bains, 1997; Frontali and Pizzi, 1999; Bultrini et al. 2003), although the meaning of such differences in terms of the linguistic metaphor of the genome has been disputed (Konopka and Martindale, 1995; Chatzidimitriou-Dreismann et al. 1996; Martindale and Konopka, 1996; Tsonis et al. 1997). String counts, after correction for underlying base composition, have been assembled into vectors known as *genome signatures*, reflecting their apparent distinctiveness between genomes (Karlin and Mrázek, 1997; Karlin et al. 1997; Karlin, 1998; Karlin et al. 1998; Campbell et al. 1999). Such composition-corrected string frequency vectors have proved useful in detecting horizontal gene transfer events between species of bacteria (Karlin, 2001). A further development based on genome signatures is that of *compositional spectra*, designed to reduce vector size and increase technical tractability (Bolshoy, 2003; Kirzhner et al. 2003).

This paper investigates the meaning of the linguistic metaphor in more detail in protein sequences, with particular emphasis on the identification of words. A protein word, rather than a string, is here taken to be more literally comparable to a word within a text of human origin. Therefore, words are only a subset of strings. Likewise, a word differs from a motif, in that motifs are often fuzzy (meaning tolerant to substitution) and are best viewed in the context of an alignment of related sequences. Within a text of human origin, a word has some context-independence. It has clear boundaries and may appear flanked by very different text in different cases. Fuzziness is also not tolerated; a word has a correct spelling. The total assembly of detected words is referred to as the *vocabulary*, and the word detection process as *vocabulary analysis*.

The pioneering vocabulary analysis in DNA sequences was carried out by Brendel et al. (1986). Their metric was based on contrasting frequencies of substrings within the candidate word. For a string, *s*, of length *k*, its expected occurrence, *E*, is the product of the occurrences of its left and right substrings, divided by the occurrence of its internal substring.

$$\begin{array}{c} E(s_{1}\ldots s_{k})=f(s_{1}\ldots s_{k-1})*f(s_{2}\ldots s_{k})\\ /f(s_{2}\ldots s_{k-1}) \end{array}$$

For each string, *s*, the difference between its expected occurrence, *E(s)* as calculated above, and actual occurrence, *f(s)*, is quantified by:

$$std(s)=(f(s)-E(s))/\text{max}\left\{\sqrt{E(s)},1\right\}$$

This provides a *z*-score for the actual occurrence of the string. Brendel et al. (1986) define a *contrast word* as any string where *std(s)* ≥ 3. Brendel et al. (1986) were able to identify several contrast words of lengths *k* = 3 to 6 in the genomes of *E. coli* and two coliphages. Conversely, avoided words could also be detected, where *std(s)* ≤ −3. An essentially similar metric has been implemented by others (Phillips et al. 1987a, 1987b; Merkl et al. 1992; Colosimo et al. 1993; Castrignanò et al. 1997; Rocha et al. 1998; Apostolico et al. 2003).

In principle, this method could also be applied to detect contrast words in protein sequences, but the combinatorial explosion caused by the presence of a 20-letter code in proteins as opposed to the 4-letter code in DNA, has restricted work on string frequency in proteins to *k* = 2 (i.e. dipeptides) only (Solovyev and Makarova, 1993). Application of the contrast words method to human texts was extended by Schmitt et al. (1996). Analysing *Alice in Wonderland*, they found that it performed relatively poorly, essentially due to the fact that the 26-letter alphabet of a text in English has a string combinatorial explosion problem even worse than that of 20-letter protein sequences.

This paper proposes improvements on the contrast words method, initially comparing their performance, in the tradition of Schmitt et al. (1996), on *Alice in Wonderland*. The most accurate method for identifying true words is then applied to several other human texts, to the NRL3D set of proteins of solved structure, and to the proteome sets of several species from all three superkingdoms (NCBI Taxonomy Browser classification) of cellular organisms.

The concept of synonymity is familiar in molecular biology. Within the degenerate genetic code, many amino acids may be encoded by more than one codon. A protein sequence may therefore be potentially coded by a combinatorially vast number of synonymous DNA sequences. Here the term is used in a more general sense. When two protein strings have different sequences, but perform the same function in their respective proteins, they are said to be *functionally synonymous*. Fuzzy motifs are an example of functional synonymity within protein families. The converse concept, that of *homonymity*, has not been explored (although see Lennon and Nussinov, 1984). Where a non-fuzzy word occurs in two different proteins and performs a different function in each, that peptide word is *functionally homonymous*. At a trivial level, it is immediately possible to see that the longer a peptide, the less likelihood it has of functional homonymity. The questions of the longest existing homonymous word, the prevalence of peptide homonymity, and its origins are all explored in this paper.

## Methods

### Texts and protein sequence sources

Public domain texts were downloaded from Project Gutenberg (http://www.gutenberg.org). Punctuation, non-alphabetic characters, numbers and spaces were removed. Word counts were case-insensitive.

The NRL3D set of sequences of proteins of solved structure (Pattabiraman et al. 1990) was downloaded from the University of Hong Kong (http://bioinfo.hku.hk/db/nrl_3d/NRL3D/nrl_3d.seq). Non-contiguous sequences (those annotated as “fragments”), sequences containing ambiguities and exact duplicates were removed using a Perl script. This reduces the number of sequences from 23301 to 6168. Further trimmings were performed using CD-HIT (Li and Godzik, 2006), which can produce datasets with maximum degrees of pairwise identity. Such reduced sets are subsequently referred to as NRL3D_*nn*, where *nn* is the maximum pairwise identity. The justification for this trimming is that most words will occur in closely related sequences, and will consequently be explicable at a trivial level. Trimming with CD-HIT reduces the number of words detected and maximises the likelihood that they will be found in less closely related proteins, and thereby be potentially more interesting from a functional point of view. As a negative control, trimmed NRL3D data sets were shuffled using shuffleseq (http://emboss.sourceforge.net/apps/release/4.0/emboss/apps/shuffleseq.html) from EMBOSS (Rice et al. 2000).

Proteomes (meaning predicted protein sets derived from genome projects) were downloaded from the EBI Integr8 database (http://www.ebi.ac.uk/integr8). They were similarly reduced by CD-HIT.

### Vocabulary analysis algorithms

For each text or proteome, and for NRL3D, overlapping strings of all lengths from *k* = 1 to 20 were counted using a Perl script running the BioPerl (Stajich et al. 2002) SeqWords module (http://doc.bioperl.org/releases/bioperl-current/bioperl-live/Bio/Tools/SeqWords.html). The SeqWords output was then analysed in the following ways. Each metric is given an acronym for easier reference.

#### 1) **CW:** Contrast words method (see Introduction)

This is the method of Brendel et al. (1986). The difference is that the *std(s)* threshold was set at 0.1 to maximise the number of candidate words.

#### 2) **RS:** Raw strings

The simplest possible method: all strings of length *k* ≥ 5, occurring at *n* ≥ 20, were assessed as candidate words.

#### 3) **ES:** Equal substrings

The raw strings extracted as above were trimmed to include only those having equal occurrences of left and right substrings.

$$f(s_{1}\ldots s_{k-1})=f(s_{2}\ldots s_{k-1})$$

The rationale for this approach is that many true words tend to satisfy this criterion. For instance, in *Alice in Wonderland*, the true word ALICE is revealed by:

f(ALIC)=f(LICE)

following to the fact that *Alice in Wonderland*, despite referring to several species, does not mention lice.

#### 4) **CW-ESM:** Equal substrings of middle substring of contrast words

Combining methods 1 and 3, middle substrings were extracted from contrast words with *std(s)* > = 0.1. These were then examined for equal substrings:

$$f(s_{2}\ldots s_{k-2})=f(s_{3}\ldots s_{k-1})$$

The rationale for this approach is the *ad hoc* empirical observation that false positive contrast words, of which there are many (Schmitt et al. 1996), frequently have true words embedded within them as middle substrings.

#### 5) **RS-ESM:** Equal substrings of middle substring of raw strings

Combining methods 2 and 3, since equality of substrings within the middle strings of contrast words was frequently found to be an indicator of a true word, the same was applied to raw strings. The additional proviso was that the left and right substrings of the raw string were not of equal occurrence to each other or the middle substring.

$$\begin{array}{ll} & f(s_{2}\ldots s_{k-2})=f(s_{3}\ldots_{k-1})\\ \text{and} & \\ & f(s_{1}\ldots s_{k-1})\neq f(s_{2}\ldots_{k})\\ \text{and} & \\ & f(s_{1}\ldots s_{k-1})\neq f(s_{2}\ldots s_{k-1})\\ \text{and} & \\ & f(s_{1}\ldots s_{k})\neq f(s_{2}\ldots s_{k-1}) \end{array}$$

The rationale for this was that, for instance, within the raw string DALICET, the true word ALICE is revealed by:

$$\begin{array}{ll} & f(ALIC)=f(LICE)\\ \text{and} & \\ & f(DALICE)\neq f(ALICET)\\ \text{and} & \\ & f(DALICE)\neq f(ALICE)\\ \text{and} & \\ & f(ALICET)\neq f(ALICE) \end{array}$$

CW-ESM and RS-ESM are equivalent, excepting that CW-ESM takes contrast words as its starting point, and RS-ESM uses raw strings. In both cases the candidate word is the middle substring, should it satisfy the criteria given.

### Measurement of accuracy

In human texts it is possible to score true words among the detected candidate words. Accuracy is measured using the Sen2 statistic (Milanesi and Rogozin, 1998):

Sen2=TP/(TP+FP)

where *TP* are those candidate words identified as true positives, and *FP* are those identified as false positives.

Perl scripts are available on request from the author.

### Assessment of hits

Protein domains were determined by reference to Pfam (http://www.sanger.ac.uk/Software/Pfam—Finn et al. 2006) and Prosite motifs detected using ScanProsite (http://www.expasy.ch/tools/scan-prosite—de Castro et al. 2006). Alignments were performed using ClustalW (Chenna et al. 2003) or bl2seq (http://www.ncbi.nlm.nih.gov/bl2seq/wblast2.cgi—Tatusova and Madden, 1999).

### Structural visualization

Solved proteins structures were downloaded from PDB (http://www.pdb.org) and visualization was carried out in MOE (http://www.chemcomp.com).

## Results

### Vocabulary analysis in human texts

*Alice in Wonderland* is a short novel of 26587 words. The total vocabulary is 2593 different words, of which 1475 are used more than once and 1072 more than twice. For illustrative purposes, the 10 commonest words are shown in Table 1. As might be expected, these are all small prepositions and pronouns, except for the name “Alice” which has 386 occurrences and is the 10th commonest word, and the verb past tense “said” at 462 occurrences.

The words in Table 1 are derived from a spaced text, with only punctuation and other extraneous characters removed. Spaces were then removed for testing of the various metrics. Again for illustrative purposes the top 10 hits using each method are shown (Tables 2 to 6), but the final comparison was made using all the hits for each method (Table 7).

#### 1) RS metric

The commonest raw strings in *Alice in Wonderland* of length *k* = 5 to 20 are tabulated in Table 2. Only 3 of the commonest raw strings in Table 2 are true *discrete words or phrases* (DWoPs—shaded grey). “Alice” as a raw string has a slightly higher occurrence than the word “Alice” in a spaced text (397 vs. 386—see Table 1) as it also occurs as part of the possessive “Alice’s”. As Table 2 suggests, RS is a relatively poor metric for identifying true words. Almost all of the raw strings in Table 2 are components of the single DWoP “said the”.

#### 2) CW metric

CW (Brendel et al. 1986) performs equally poorly, as previously demonstrated by Schmitt et al. (1996). Table 3 shows the top 10 contrast words of length *k* = 7 to 20, sorted by descending *std(s)*. There are only two DWoPs detected.

It was noted that the some of the false positive contrast words in Table 3 contained the true DWoPs “of the” (twice), “in the” and “little” as their middle substrings. This stimulated the further investigation of the middle subwords.

#### 3) ES metric

Table 4 tabulates the 10 highest hits with ES, sorted by their occurrence, *n*. This contains 6 true DWoPs (shaded).

ES performs rather better than CW or RS, although it can accumulate nested strings. For instance in Table 4, “saidalic” is found to be a substring of “saidalice”, “littl” of “little” and “heking” of “theking”. This suggested the combination of ES with the other methods.

#### 4) RS-ESM metric

RS-ESM shows a further marked improvement. Nested substrings are avoided, and 9 out of the top 10 hits are true positives (Table 5).

#### 5) CW-ESM

CW-ESM appears to be the best method on first examination. All of the top 10 hits are true DWoPs (Table 6). However, a decision on the best method to apply to biological sequences requires a fuller assessment of the output beyond the top 10 hits.

### Comparison of methods

Table 7 compares the methods on *Alice in Wonderland*. Since the initial string count was to *k* = 20, the two ESM methods are limited to *k* = 18 as their longest identifiable word.

Table 7 demonstrates that the CW metric is the poorest. Although it generates a large number of hits, the true positive rate is barely more than 6%. RS gives greater numbers of candidate words as the thresholds for occurrence and string length are dropped, but Sen2 does not rise above 24%. Adding a requirement for equal right and left substrings, ES, brings the number of candidates down dramatically—from 1213 hits to 241 hits where *k* = 5–20, *n* ≥ 0. Sen2 increases from 17% to just over 25%. However, for the combination methods, Sen2 increases considerably. For contrast words (CW-ESM) just under 58% accuracy can be achieved, and just over 68% accuracy for raw strings (RS-ESM). The latter also has a larger number of hits, generating 895 true positives. Considering only 1042 words are used more than twice in *Alice in Wonderland*, this is a reasonable figure.

**Figure 1.**
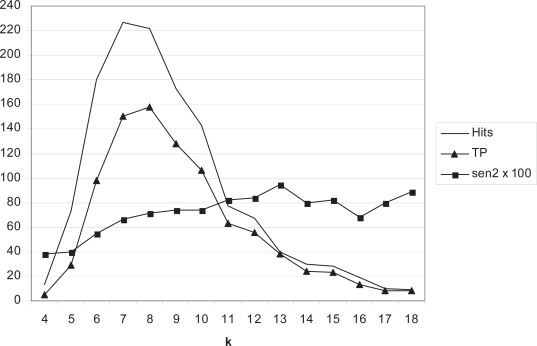
RS-ESM performed on *Alice in Wonderland*. Number of hits plotted against *k*. TP: true positives. Sen2 is also plotted (×100) to show its improvement at higher levels of *k*.

The next question to be investigated is whether or not the quality of hits varies across *k*. Figure 1 plots the true positive rate against the length of the candidate word for RS-ESM in *Alice in Wonderland*. Sen2 increases with length *k*. Although the overall Sen2 is 0.682 (Table 7), Sen2 rises above 0.8 for *k* = 11–15. The majority of strings of length *k* = 4 and 5 are false positives.

**Figure 2.**
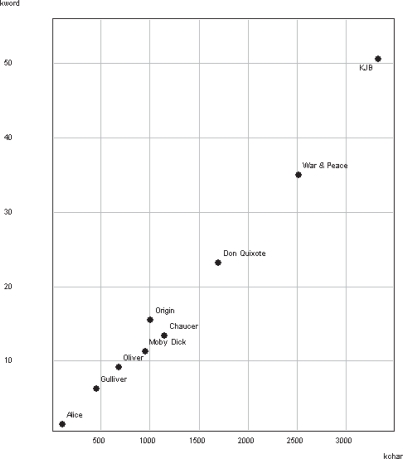
Number of candidate DWoPs (×1000, kword) plotted against length of text (kchar) for RS-ESM, *k* = 6–18. Alice: *Alice in Wonderland*, Gulliver: *Gulliver’s Travels*, Oliver: *Oliver Twist*, Chaucer: *Canterbury Tales* in 19th century translation, Origin: *Origin of Species*, Don Quixote: 19th century English translation of same, KJB: *King James Bible*.

Extending the analysis to a range of other human texts, Figure 2 plots the number of candidate DWoPs detected for each against the length of the text in 1000s of characters (kchar). There is a clear correlation (*r* = 0.994) between number of different words and size of text. This has also been observed for the number of different raw strings, a phenomenon known as Heaps’ Law (Heaps, 1978).

### Vocabulary analysis in sets of real and shuffled protein sequences

**Figure 3.**
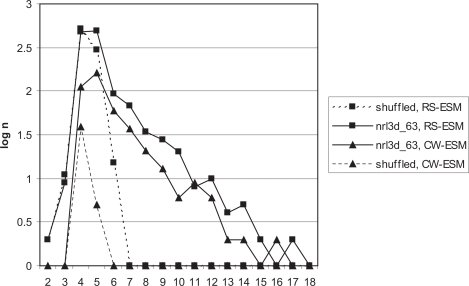
NRL3D_63 (solid lines) and its shuffled equivalent (dotted lines), tested with both RS-ESM (squares) and CW-ESM (triangles). The logarithm of the number of hits, *n*, is plotted against *k*. Log(0) is arbitrarily designated zero. Pseudocounts are therefore added when *n* = 1.

Figure 1 suggests that there may be increased artefactual detection of false positive DWoPs for RS-ESM at *k* = 4 and 5, based on the identification of such false positives in *Alice in Wonderland*. Greater than 60% true positivity is only obtained at *k* ≥ 7 and 80% at *k* ≥ 11. When a text of human origin is being analysed, one can reliably identify the false positives and thus precisely quantify Sen2. However, in a protein sequence set, whether NRL3D or a naturally occurring proteome, scoring of accuracy requires the use of shuffled sequences. In the shuffled sequences, all hits are by definition artefactual. Figure 3 plots the distribution of candidate words in real and shuffled NRL3D_63 protein sequence set (see Methods) for both RS-ESM and CW-ESM methods. It can be seen that the shuffled sequence sets give false positives at up to *k* = 6 for RS-ESM. However, the ratio of hits of *k* = 6 in the real as compared to the shuffled genome is much higher than at *k* = 5 or less. Therefore, it seems that *k* = 6 should be considered an ambiguous category. Although most hits at *k* = 6 are likely to be genuine, there is a far greater risk of a false positive than at *k* ≥ 7. The observation that Sen2 is less than 0.5 for *k* ≤ 5 (Fig. 1) also justifies concentration on longer candidate words. This supports the earlier finding by Thode et al. (1996), who found that matches of 6 residues within a window of length 10, could be found far more frequently between pairs of real proteins than random sequences. By contrast, CW-ESM, although producing fewer hits, has no hits in shuffled sequences above *k* = 5. Therefore, it might be preferred for investigating words of length *k* = 6.

### Structural meaning of words

The words of *k* = 12–18 identified in NRL3D_63 using RS-ESM are shown in Table 8.

The protein family in which the word is located is designated from the NRL3D annotation, or where that is ambiguous, by reference to Pfam (Finn et al. 2006). Most of these hits are found within fairly well conserved proteins, often orthologues having the same essential function in different species within the same major phylogenetic class. In some cases, however, the hits are found to be stretches of total conservation within otherwise somewhat divergent proteins, often having slightly variant functions and from rather more distant species. The 14-mer LGGTCVNVGCVPKK is found in glutathione reductase (EC 1.6.4.2) from humans and *E. coli*, and in the related enzyme trypanothione reductase (EC 1.6.4.8) from two genera of trypanosome.

**Figure 4.**
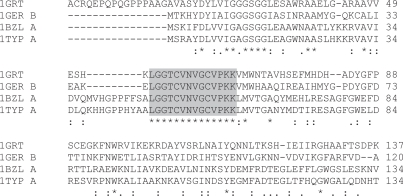
The first 150 residues of the alignment of the four sequences containing the word LGGTCVNVGCVPKK (shaded). The proteins are identified by their PDB designations—1GRT: human glutathione reductase; 1GER: *E. coli* glutathione reductase; 1BZL: *Trypanosoma cruzi* trypanothione reductase; 1TYP: *Crithidia fasciculata* trypanothione reductase.

**Figure 5.**
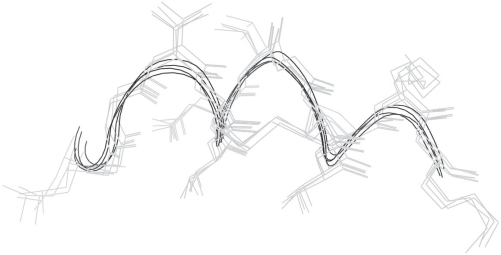
Superposition of sequence LGGTCVNVGCVPKK in the 4 proteins aligned in Figure 4. The helical backbone is shown in black. Despite the variability of the other parts of these proteins, LGGTCVNVGCVPKK represents a region of extreme structural, and presumably functional, conservation between *E. coli*, humans and trypanosomes.

Although LGGTCVNVGCVPKK is completely conserved within an alignment having generally poor levels of conservation (Fig. 4), spanning bacteria, trypanosomes and humans, all these proteins possess a pyridine nucleotide-disulphide oxidoreductase domain (Pfam PF07992). LG-GTCVNVGCVPKK is also recognised by Scan-Prosite (de Castro et al. 2006) as containing a pyridine_redox_1 motif (ProSite PS00076). The word therefore may be taken to have equivalent function within these proteins and is not a homonym as defined in the Introduction. LGGTCVN-VGCVPKK in all four cases is found at the beginning of a long helix. Superposition of the structures of the 4 proteins to 1.894 Å in MOE demonstrates excellent conservation even over the poorly conserved regions. LGGTCVNVGCVPKK assumes a highly similar structure in all cases (Fig. 5).

**Figure 6.**
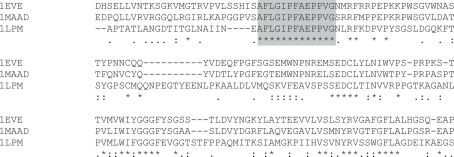
The first 150 residues of the alignment of the three sequences containing the word AFLGIPFAEPPVG (shaded). 1EVE: *Torpedo californica* acetylcholinesterase; 1MAAD: mouse acetylcholinesterase chain D; 1LPM: *Candida rugosa* lipase.

As an additional example, AFLGIPFAEPPVG is found in the N-terminal regions of acetylcholin-esterase (EC 3.1.1.7) from mouse and the electric ray and also in triacylglycerol lipase (EC 3.1.1.3) from yeast. As before, the word represents a stretch of total conservation in an otherwise low identity alignment (Fig. 6). Despite this, all 3 of these proteins contain a carboxylesterase domain (Pfam PF00135), and their solved structures may be superposed over their full length to 3.70 Å in MOE (not shown).

One phenomenon that appears in the output, that has no analogue in texts of human origin, is the detection of homopolymers. The longest homopolymeric word in NRL3D_63 is the heptamer AAAAAAA, detected in antifreeze protein A from the flounder and also in an amine dehydrogenase from *Thiobacillus versutus*. However, it occurs in the extreme C-terminus and N-terminus respectively of these two proteins. Homopolymers are a consequence of regions of low complexity within coding DNA, and have no analogue within human texts. They formally constitute words, and indeed in the case of AAAAAAA a homonym, within the terms of the algorithms used, but are neglected owing to their lack of likely functional significance.

**Figure 7.**
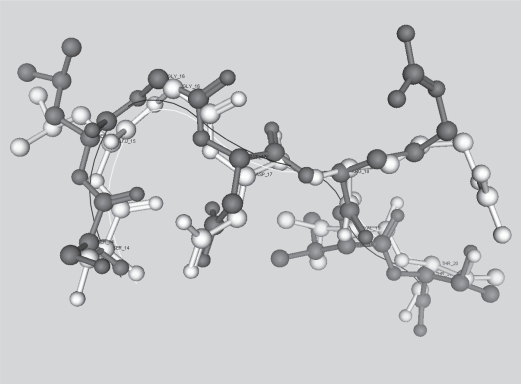
Superposition of SLGDRVT in mouse antibody proteins 1JRHL (white) and 1NMCL (black). Backbone traces are rendered as fine lines.

Leaving aside the homopolymers, there is only one identifiable homonym in NRL3D_63 of *k* ≥ 7. SLGDRVT is found in a beta-lactamase from *Streptomyces albus* and also in two mouse antibody proteins (1JRHL and 1NMCL). The two mouse proteins are 61% identical as assessed by bl2seq (Tatusova and Madden, 1999), and SLGDRVT is found in both cases in the N-terminus of the solved structure of the protein, where it is part of the V-set domain (Pfam PF07686) The two mouse proteins superpose to 0.821 Å over the entire length of their solved structures (not shown), and their SLGDRVT sequences have good structural alignment of their backbones (Fig. 7).

**Figure 8.**
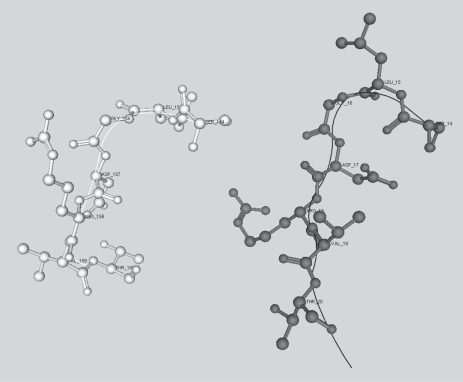
Comparison of SLGDRVT word in *Streptomyces albus* lactamase 1BSG (white) and mouse antibody protein 1NMCL (black). Backbone traces are rendered as fine lines.

In the eubacterial lactamase 1BSG, SLGDRVT is found in a different conformation (Fig. 8). In this protein is it part of a helix rich beta-lactamase domain, but does not occur within a helix.

SLGDRVT is the only homonym detectable in NRL3D_63 at *k* = 7 using RS-ESM. Although there are many at *k* = 6 (37 with CW-ESM and 36 with RS-ESM). As shown in Figure 3, CW-ESM may be preferable to RS-ESM at *k* = 6 in that, although less sensitive, it is less inclined to false positives at *k* = 6.

In summary, within NRL3D_63, longer words are mostly indicative of conservation. Some of them are islands of ultra-conservation within distinctly divergent proteins. However, annotation or Pfam domain mapping indicates that these are always, at least in the cases examined (both above and data not shown), within proteins of similar general functionality. The longest homomym is a solitary example found at *k* = 7 but they appear to be plentiful at *k* = 6. The latter however, must be under suspicion of false positivity, owing to the number of hits at *k* = 6 in shuffled versions of the NRL3D database. The relative paucity of homonyms of reliable length suggests that future fine-tuning of the algorithm ought to be performed on protein sequence sets where functional annotation of motifs and domains is more complete than in NRL3D.

Since NRL3D is a compendium of proteins of highly diverse origin, but also enriched for sequences of easily solved structure, its vocabulary may be very different in character to that of individual proteomes. These were therefore examined for the presence of homonyms and island of extreme conservation.

### Vocabulary analysis on individual proteomes

**Figure 9.**
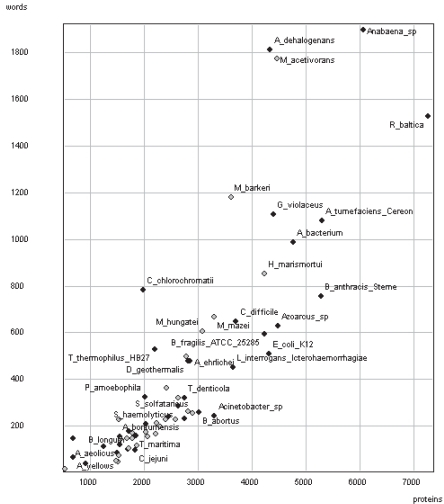
Candidate words of *k* = 6 to 18, detected using RS-ESM, against number of proteins for 35 eubacterial (black circles) and 28 archaeal (grey circles) proteomes.

Figure 9 plots the number of words detected using RS-ESM versus the size of the proteome in terms of number of proteins. All proteomes were previously reduced to no more than 63% identity by use of CD-HIT, as performed on NLR3D. Figure 9 indicates that Heaps’ Law (see Fig. 2 above) also applies to proteomes. This had previously been observed for raw strings in proteins (Mukhopadhyay et al. 2006). The same trend applies when the proteomes are measured in kilo-residues (comparison not shown).

Figure 9 shows the same general relationship for proteomes as is demonstrated in Figure 2 for texts. The correlation is weaker for eukaryotes (not shown in Fig. 9) and archaea (*r* = 0.905 and 0.907 respectively), but comparable for eubacteria (*r* = 0.996 against *r* = 0.994 for texts). However, the range of proteome size in eukaryotes is generally not comparable with the other two superkingdoms, making it difficult to draw any conclusions concerning differences in vocabulary structure between super-kingdoms. Supplementary Material Tables 1, 2 and 3 give the full results for the various species.

Table 9 shows that texts of human origin have a far richer vocabulary than proteomes, and that eukaryotes appear to have a richer vocabulary than eubacteria or archaea. However, when only eukaryotic proteomes within the size range of the other two kingdoms are considered, this discrepancy decreases markedly, suggesting that it should be interpreted with caution.

Detailed analysis of all proteome sets would be inappropriate for a single paper. A number of individual proteomes were chosen for further analysis, contrasting the three superkingdoms, and also small and large proteomes where possible.

### Vocabulary analysis in a small eubacterial proteome

*Chlamydia muridarum* has 916 proteins, of which 914 are no more than 63% identical, indicating a virtual absence of gene families of closely related proteins. Using RS-ESM, *C. muridarum* contains 34 words of which 17 are *k* ≥ 7 (Supplementary Material Table 4). One of these is the homoheptamer DDDDDDD, and 7 others are words that occur several times within single proteins, indicating repetitive sequences, or occurring in low complexity areas of proteins. Of the remainder, all fall within clearly related proteins, except for two. The first of these, GPTGSGK appears at first glance to constitute a homonym, occurring in 4 proteins (SwissProt identifiers Q9PLF7, Q9PLM1, Q9PJG9 and Q9PKD0) with different Pfam domains, although all are ATP-binding proteins. In each case GPTGSGK is found at or near the N-terminus of the main Pfam domain within the protein (being IPPT, AAA_2, GSPII_E and ABC_tran respectively, listed as “various” in Supplementary Material Table 4). In the case of Q9PKD0, GPTGSGK is annotated by ScanProsite as an NP_BIND ATP binding motif. Therefore, GPTGSGK is probably not a homonym but rather an ATP-binding cassette conserved or convergently evolved across divergent proteins within *C. muridarum*.

**Figure 10.**
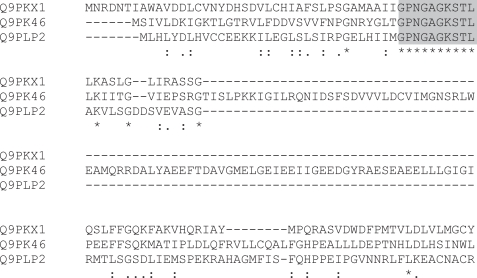
N-terminal region of 3 *C. muridarum* transporter proteins showing the GPNGAGKSTL word (shaded).

A second word of interest is an ultra-conserved region within a set of rather divergent transporter proteins, where it constitutes, again like GPTGSGK, the NP_BIND motif for ATP-binding (Fig. 10). GPNGAGKSTL and GPTGSGK can be represented by the profile GP(T/N)G(A/S)GK.

Of the 17 words in *C. muridarum* of *k* = 6, all but 4 appear to be homonyms. However, these must be regarded with suspicion, as they can occur artefactually at *k* = 6 in shuffled sequences (Fig. 3). When *C. muridarum* is examined with the less sensitive CW-ESM algorithm, which is also less liable to artefactual hits at *k* = 6, there are only 6 hits at that length, of which only one is a homonym.

### Vocabulary analysis in a large archaeal proteome

*Methanosarcina acetivorans* has 4467 proteins, of which 4080 are no more than 63% identical, indicating that around 10% of the total is comprised of members of moderately or closely related protein families, in contrast to the virtual absence of such families in *C. muridarum*. In order to make the analysis more tractable, the *M. acetivorans* proteome is first trimmed to 40% maximum identity, reducing it to 3655 proteins. Using RS-ESM, *M. acetivorans* contains 946 words or which 659 are *k* ≥ 7 and 300 are *k* ≥ 10. Those satisfying both *k* ≥ 10 and *n* ≥ 9 are shown in Supplementary Material Table 5.

**Figure 11.**
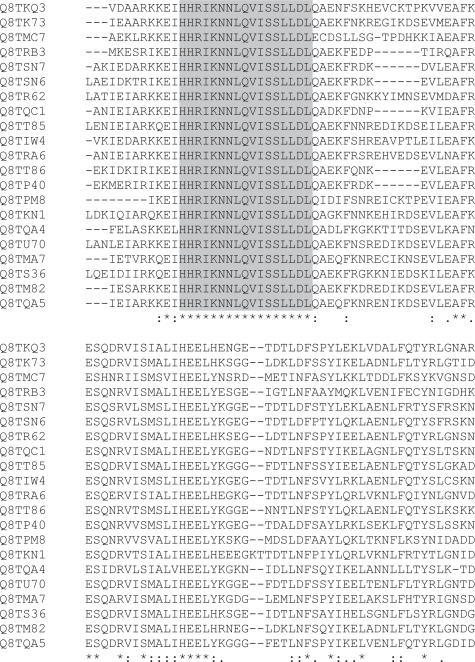
HHRIKNNLQVISSLLDL (shaded) in part of a histidine kinase alignment.

The words mostly represent islands of extreme conservation within what are fairly divergent families. For instance the 17-mer HHRIKNNLQ-VISSLLDL is found in histidine kinases (Fig. 11), where its location corresponds to the start of the HisKA_2 domain (Pfam PF07568). There do not appear to be any homonymous words of *k* ≥ 10 in the *M. acetivorans* proteome. *M. acetivorans* words are dominated by the preponderance of components of histidine kinase domains and PKD domains (Pfam PF00801).

### Vocabulary analysis in a medium-sized eukaryotic proteome

The fungus *Yarrowia lipolytica* has 6524 proteins of which 5864 are <40% identical. It therefore has almost exactly the same overall proportion (just under 90%) of proteins in gene families as *M. acetivorans*. Using RS-ESM, *Y. lipolytica* contains 1954 words of which 940 are *k* ≥ 7 and 401 are *k* ≥ 10. All words satisfying both *k* ≥ 10 and *n* ≥ 9 are shown in Supplementary Material Table 6. By contrast with *M. acetivorans*, the prominent *Y. lipolytica* words are composed entirely of simple sequence repeats.

### Vocabulary analysis in a large eukaryotic proteome

*Brachydanio rerio*, the zebrafish, has 14049 proteins, of which 8312 are no more than 40% identical, indicating that just over 40% of the total are members of moderately or closely related protein families, a considerably higher proportion than in the smaller eukaryotic proteomes (at ~10% for *Y. lipolytica*). Using RS-ESM, *B. rerio* contains 2938 words or which 1380 are *k* ≥ 7 and 452 are *k* ≥ 10. All words satisfying both *k* ≥ 10 and *n* ≥ 9 are shown in Supplementary Material Table 7.

Just as prominent *M. acetivorans* words are dominated by components of histidine kinase domains and PKD domains, prominent *B. rerio* words are in most cases part of an EGF domain, with a handful of SCRC domains (PF00530). There are also several examples of low complexity words (Supplementary Material Table 7), similar to *Y. lipoloytica.*

## Discussion

An improved algorithm for vocabulary analysis in texts of human origin, has been applied to proteomes. In its two variants, CW-ESM and RS-ESM, it achieves an accuracy of 60%–70% (Table 7) and in the case of RS-RSM has approximately 85% sensitivity. This sensitivity estimate is based on 895 true positive hits as compared to the 1042 words used more than twice in *Alice in Wonderland*. It remains an approximation as the algorithm detects phrases longer than single words (DWoPs, see Tables 2–6). Although CW-ESM is slightly less accurate than RS-ESM and less than half as sensitive (Table 7), it is less liable to false positives at *k* = 6 (Fig. 3). Since many protein homonyms appear at *k* = 6, CW-ESM remains an important accessory algorithm for the study of short words in protein sequences. This paper therefore solves the problem posed by Schmitt et al. (1996) of how to apply the method of Brendel et al. (1986) to longer alphabets. Since the combinatorial explosion problem is greater in human texts than in protein sequences, the adequacy of the algorithm for detecting words in texts implies that it can do the same for proteins, should such words exist.

It is notable that the words detected by the algorithm follow Heaps’ Law, a linear increase in word count as text size increases, for both human texts (Fig. 2) and proteins (Fig. 9). A similar result for raw strings in proteins is already known (Mukhopadhyay et al. 2006). Within superkingdoms, Heaps’ Law correlations are strongest for human texts and eubacterial proteomes. By contrast, between superkingdoms, eukaryotic proteomes appear to be nearly three times more word-rich on average than the two prokaryotic superkingdoms. However, caution must be exercised in inter-superkingdom comparisons as the average proteome size is almost four times larger for eukaryotes. When only small eukaryote proteomes are used, the proportionately larger number of words decreases to similar levels (Table 9). Heaps’ Law therefore appears to deviate from linearity in large eukaryotic proteomes, but eukaryotic proteomes may still be comparable to prokaryotic proteomes at smaller sizes. One possible explanation for this is that larger eukaryotic proteomes are richer in gene families, adding an extra source of words to the general trend implied by Heaps’ Law, and supported by the observation that about 40% of *B. rerio* proteins are >40% identical.

It should be noted that vocabulary analysis is not the same as segmentation (Wang, 2001; Cohen et al. 2002), when a text known to be composed of words is split into candidate words. Segmentation is often used in computer analysis of pictographic languages such as Japanese *kanji* script, where word boundaries are unclear. By contrast, vocabulary analysis algorithms search for the presence of candidate word structures in bodies of symbols that may not necessarily contain them.

The fact that human texts are an order of magnitude more enriched in words than proteomes (Table 9), suggests that the linguistic analogy for biological sequences remains a weak one, and furthermore that segmentation algorithms, relying as they do on complete decomposition of the text into words, are unlikely to be applicable to protein sequences. Nevertheless, the presence of identifiable word-like structures within proteomes is intriguing. Shuffled proteomes, like shuffled texts, lose their word content. Within a shuffled proteome, false positives are rare and in neither RS-ESM nor CW-ESM are found at *k* ≥ 7. Words of *k* = 6 are ambiguous, as they are generated as false positives by RS-ESM (Fig. 3). By analogy with words in human texts, proteome words are suggested to be sequences that are intolerant to mutation but are nevertheless relatively context-independent in their function.

Analysis of the distribution of words in individual proteomes demonstrates two main categories:

1. conserved stretches within proteins of essentially similar function (see Figs. 4–6).
2. homonyms appearing in proteins of demonstrably different functions (see Fig. 8).

Conserved words can be further split into:

- relatively uninteresting sequence identities within closely related proteins
- ultra-conserved words in rather more divergent proteins (see Figs. 4, 5, 6, 10 and 11 for examples).

Homonyms are plentiful but short, rarely *k* > 6, whereas ultra-conserved stretches are often much longer, for instance the 17-mer HHRIKNNLQ-VISSLLDL (Supplementary Material Table 5 and Fig. 11) which forms a word in a family of histidine kinase proteins in *M. acetivorans*. Only words of up to *k* = 18 were tested in this paper, so no estimate can be made of the longest existing word. *M. acetivorans* has a low complexity 18-mer, STDDSTDDSTDDSTDDST (Supplementary Material Table 5), and *Y. lipolytica* has eight (Supplementary Material Table 6). The longest high complexity words are the 6 EGF domain words and a zinc finger word found in *B. rerio* (Supplementary Material Table 7).

Not all words can be easily designated as homonyms or conserved. For instance, in *C. muridarum* GPTGSGK is found in different Pfam domains in different proteins (IPPT, AAA_2, GSPII_E and ABC_tran), initially suggesting homonymity. However, in all these cases GPTGSGK forms part of an ATP-binding cassette. Whether this is best explained by ultra-conservation within highly divergent ATP-binding proteins with a distant GPTGSGK-containing ancestor or by the multiple evolution of ATP binding capacities by convergence to these words, is debatable. The similar longer word GPNGAGKSTL (Fig. 10) is also an ATP-binding element, but, unlike GPTGSGK, GPNGAGKSTL is always found in the ABC_tran domain (PF00005), and therefore is more likely to be an example of ultra-conservation than convergence.

**Figure 12.**
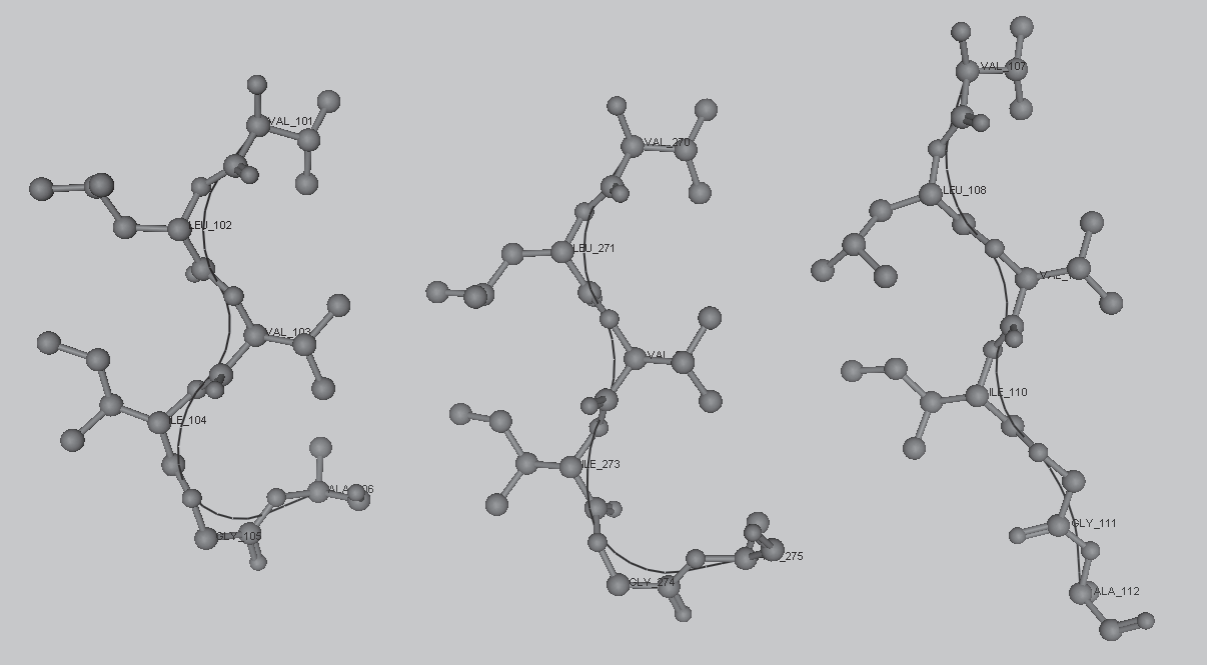
VLVIGA in (from left to right) 1D4O.A, 1BFD and 1AD3.A. The backbone trace is drawn as a black line.

Homonyms are assumed to have different functions within their respective proteins, especially when they can be shown to have different structures (e.g. see Fig. 8). However, apparent homonyms with similar structures may be a result of convergent molecular evolution on a micro-scale, perhaps the case with *C. muridarum* GPTGSGK. For a specific structural example, VLVIGA is a 6-mer homonym in NRL3D, occurring in: 1BFD, a benzoylformate decarboxylase (EC 4.1.1.7) from *Pseudomonas putida*; 1AD3A, an aldehyde dehydrogenase (EC 1.2.1.5) from rat; and in 1D4OA, a bovine NADPH transhydrogenase (EC 1.6.1.1). In each of these cases VLVIGA is found in a different Pfam domain: in TPP_enz_M (PF00295), Aldedh (PF00171) and PNTB (PF02233) respectively. Nevertheless, the conformation of VLVIGA is remarkable similar in each case, always being the point at which a short beta-sheet ends (Fig. 12).

Comparison of different proteomes indicates that the peptide vocabularies can be quite different in character from species to species. For instance, the prominent words in the small eubacterial species *C. muridarum* are dominated by components of adherence factor proteins mixed with a handful of peptides from other domains and some low complexity elements (Supplementary Material Table 4). The large archaeon *M. acetivorans* has many words from PKD and histidine kinase domains (Supplementary Material Table 5). The fungus *Y. lipolytica* has only low complexity words within its major vocabulary (Supplementary Material Table 6). Finally, the vertebrate *B. rerio* is dominated by EGF domain words (Supplementary Material Table 7). A fuller exploration of how typical these vocabulary patterns may be, is beyond the scope of the present paper. However, it indicates that each proteome may potentially be identifiable by the characteristics of its vocabulary; e.g. rich in low complexity, or with certain typical domain-linked vocabularies, raising the prospect of a *peptide vocabulary signature* analogous to the genome signature found in DNA. This may be useful in metagenomic analysis.

Thode et al. (1996), in pairwise comparisons of proteins, found many matches of 6 residues within windows of 10, and showed that these occurred far less frequently between pairs of random proteins. The method of Thode et al. (1996), differs from the one presented here in that they used a criterion of 60% identity with strings of *k* = 10. Unlike the present method, there was no previous algorithmic identification of candidate words by statistical properties. Rather, they commenced with a small group of proteins and extracted all their initial 10-mer strings from those sequences. These were then compared against the whole protein database, and matches of 6/10 or better recorded. It thus has some similarities to method RS above, but incorporating fuzziness. Regardless of these methodological incongruences, the detection by Thode et al. (1996) of a far greater quantity of short common strings in real protein pairs than in shuffled ones, parallels the results presented in Figure 3. Therefore, it is justifiable to believe that even words of *k* = 6 may be mostly due to something other than random coincidence. The nature of this pressure may be conservation, amply demonstrated by the various ultra-conserved words within fairly divergent proteins (Figs. 4, 5, 6 and 11) or it may be convergent evolution. The latter of these raises the possibility than the presence of an apparent homonym within a protein may imply positive selection within the family to which that protein belongs, and which may be detectable using appropriate methods (Yang, 1997; Anisimova and Yang, 2007). For instance, if a candidate homonymic word is found in two proteins of differing function, for instance different Pfam families, and positive selection can be statistically demonstrated in each of those families over the region of the homonym, a selective convergent scenario for the origin of that homonym would be highly suggestive.

## Supplement Material

**Table S1.** Words detected in eukaryotic proteomes using RS-ESM, *k* = 6–18.

**Species**	**Proteins**	**Kres**	**Words**
H. sapiens	37993	16405.3	30360
M. musculus	32971	14645.0	29463
A. thaliana	34712	14124.8	47516
T. nigroviridis	27836	11286.0	26742
C. elegans	22434	9699.5	14167
D. melanogaster	14396	8055.1	7509
D. discoideum	13017	6817.5	16628
A. gambiae	15145	6125.0	6509
C. briggsae	13192	6038.7	6687
G. zeae	11636	5952.6	5302
B. rerio	14049	5940.9	12267
A. oryzae	12053	5410.2	5498
R. norvegicus	11839	5350.2	10466
L. major	8010	5137.4	4795
D. pseudoobscura	9877	5115.2	4412
A. fumigatus	9906	4782.5	3891
P. falciparum (3D7)	5282	4001.3	10494
C. neoformans	6569	3558.9	2787
C. neoformans (JEC21)	6437	3449.1	2461
P. yoelii	7590	3385.6	9444
Y. lipolytica	6524	3118.5	3661
D. hansenii	6309	2902.5	2401
S. cerevisiae	5800	2891.7	2227
B. taurus	8292	2890.4	3652
C. glabrata	5180	2610.3	1742
K. lactis	5326	2504.6	1249
G. gallus	5387	2443.6	3384
S. pombe	5011	2351.3	1306
A. gossypii	4720	2314.3	1103
T. annulata	3790	2025.0	3409
T. parva	4070	1895.3	1899
C. hominis	3886	1757.6	924
E. cuniculi	1909	693.5	308
T. gondii	489	377.9	230
G. theta	598	178.5	38

**Table S2.** Words detected in eubacterial proteomes using RS-ESM, *k* = 6–18.

**Species**	**Proteins**	**Kres**	**Words**
R. baltica	7271	2290.5	1658
Anabaena. sp	6069	1955.5	2196
A. tumefaciens (Cereon)	5305	1687.1	1195
A. bacterium	4771	1677.5	1011
B. fragilis (ATCC 25285)	4234	1537.1	841
A. dehalogenans	4345	1516.6	1902
B. anthracis (Sterne)	5288	1460.1	996
Azoarcus. Sp.	4490	1393.5	764
G. violaceus	4406	1377.7	1412
E. coli (K12)	4323	1372.0	687
C. difficile	3711	1164.1	884
L. interrogans (icterohaemorrhagiae)	3654	1150.7	705
Acinetobacter. Sp.	3310	1048.4	266
A. ehrlichei	2862	984.1	524
A. borkumensis	2752	908.2	236
D. geothermalis	2821	901.0	538
B. abortus	3023	877.9	273
T. denticola	2753	863.6	573
S. elongatus	2451	770.0	378
S. haemolyticus	2634	756.6	370
C. chlorochromatii	1991	750.9	907
T. thermophilus (HB27)	2200	667.6	552
P. amoebophila	2023	658.9	1210
F. nucleatum	2046	641.1	250
B. longum	1723	638.8	181
T. maritima	1852	582.8	191
C. jejuni	1836	538.6	104
H. pylori (26695)	1551	491.8	172
A. aeolicus	1552	488.9	121
P. marinus (CCMP 1378)	1707	484.4	105
D. ethenogenes	1502	416.5	95
B. afzelii	1257	357.8	223
C. muridarum	916	324.3	40
M. pneumoniae	687	239.7	380
A. yellows	690	176.6	266

**Table S3.** Words detected in archaeal proteomes, using RS-ESM, *k* = 6–18.

**Species**	**Proteins**	**kres**	**Words**
M. acetivorans	4467	1392.1	2317
H. marismortui	4234	1200.1	1006
M. barkeri	3616	1126.3	1701
M. mazei	3302	1004.3	939
M. hungatei	3095	997.2	792
S. solfataricus	2910	827.9	823
N. pharaonis	2784	815.9	571
H. walsbyi	2644	787.4	387
S. tokodaii	2816	757.9	399
H. salinarium	2426	680.0	449
M. burtonii	2242	676.7	313
A. fulgidus	2398	660.8	262
P. aerophilum	2589	654.9	473
P. kodakaraensis	2301	637.7	219
S. acidocaldarius	2221	631.7	188
P. furiosus	2045	577.7	202
P. horikoshii	2077	569.4	159
P. abyssi	1785	539.2	180
M. thermoautotrophicum	1869	524.7	145
M. jannaschii	1782	504.9	213
M. kandleri	1687	501.0	205
M. stadtmanae	1533	493.6	277
M. maripaludis	1722	490.7	113
A. pernix	1576	482.7	143
P. torridus	1535	471.3	79
T. acidophilum	1482	453.2	49
T. volcanium	1523	452.7	56
N. equitans	536	151.5	17

**Table S4.** All words of *k* ≥ 6 detected in *C. muridarum* (proteins <63% identical), using RS-ESM.

**Word**	***k***	**Protein family**	***n***
GGKGGLTVQIGG	12	adherence factor	3
FQEEHGHCRVP	11	helicase	4
GPNGAGKSTL	10	ABC transporter	3
EPAPEPAPE	9	low complexity	3
SDTESTNGN	9	low complexity	3
NPQLASWV	8	helicase	4
SGSGKSSL	8	ABC transporter	3
IHDVEQNG	8	DUF1547 (PF07577)	3
GPTGSGK	7	various	4
FRVTDPN	7	adherence factor	3
GIEGLIH	7	S1 RNA binding	3
DDDDDDD	7	low complexity	3
EGRCMGL	7	adherence factor	3
NDVTPAD	7	adherence factor	3
KTAAKKA	7	histone-like	3
LGGGAIL	7	Chlam_PMP (PF02415)	3
HGIWIAG	7	adherence factor	3
SSSSSS	6	low complexity	5
RSLLNK	6	homonym	4
VLLGLG	6	homonym	4
GKLSED	6	helicase	4
SFRAIP	6	adherence factor	3
LPLFSL	6	homonym	3
SSSFAL	6	homonym	3
IAILLS	6	homonym	3
RLKTIL	6	homonym	3
ALGIAA	6	homonym	3
VVLFDE	6	homonym	3
AASLIR	6	homonym	3
SLQEGL	6	homonym	3
ALPGVG	6	homonym	3
PNVGKS	6	MMR_HSR1 (PF01926)	3
EKILSL	6	homonym	3
VLSYEL	6	homonym	3

**Table S5.** All words of [*k*≥ 10 AND *n*≥ 9] in *M. acetivorans* (proteins <40% identical), sorted by occurrence, *n*.

**Word**	***k***	**Protein family**	***n***
STDDSTDDSTDDSTDDST	18	low complexity	27
EIHHRIKNNLQVISSLL	17	histidine kinase	27
HHRIKNNLQVISSLLDL	17	histidine kinase	21
NMPVEYFDFNGN	12	PKD domain	20
VAYFHNMDWIE	11	PKD domain	20
GDGLYEDLTGNGEFSFVD	18	PKD domain	19
DLDGDGLYEDLTG	13	PKD domain	17
VVLATLTVSGKEKGSAN	17	PKD domain	15
VSGKEKGSANLSIGV	15	PKD domain	15
ISSLLDLQAEKF	12	histidine kinase	14
PLGIIVNELVSNSLKHAF	18	histidine kinase	13
GSANLSIGVKRLE	13	PKD domain	13
YSFLPVYSFLPVYSFLPV	18	low complexity	12
EGAADVVLATLTVSGKE	17	PKD domain	11
TVPEENITVPEEN	13	low complexity	11
AVPLGIIVNELVSNSLK	17	histidine kinase	10
GTAPLTVNFTDQSTGSP	17	PKD domain	9
STGSPTSWFWDFGDG	15	PKD domain	9
VSEASGSTVTLYFDP	15	PKD domain	9
PTSWFWDFGDGANST	15	PKD domain	9
LSPLPDQEYAPKDL	14	PKD domain	9
DITERKKAEEAL	12	histidine kinase	9
MDTAVPLGII	10	histidine kinase	9

**Table S6.** All words of [*k* ≥ 10 AND *n* ≥ 9] in *Y. lipolytica* (proteins <40% identical), sorted by length, *k*.

**Word**	**Protein family**	***k***	***n***
QQQQQQQQQQQQQQQQQQ	low complexity	18	67
ATDTGATATDTGATATDT	low complexity	18	22
TVTGPTAGTTTITGTDGK	low complexity	18	15
SYSPTSPSYSPTSPSYSP	low complexity	18	14
IFIFIFIFIFIFIFIFIF	low complexity	18	14
YDSYDSYDSYDSYDSYDS	low complexity	18	11
PLAEPMPLPLAEPMPLPL	low complexity	18	10
SGSGSSGSGSSGSGSSGS	low complexity	18	9
ATDTATDTAATDTATDT	low complexity	17	31
GSGSGSGSEGSGSGSGS	low complexity	17	19
SQSQSQSQSQSQSQSQS	low complexity	17	17
NGNGSDGSNGNGSDGSN	low complexity	17	16
GSGSGSGSDSGSGSGSG	low complexity	17	13
SSSIPTGDVSSATPTGD	low complexity	17	11
DASSSIPTGDVSSATPT	low complexity	17	11
PTGDVSSATPTGDASSS	low complexity	17	10
TGGADASSTGGADASST	low complexity	17	10
TATDTGATDTATDTGAT	low complexity	17	9
TEQITVAPTGPVTTKTV	low complexity	17	9
KQKQKQKQKQKQKQKQK	low complexity	17	9
ATQTGGNGNNSGSNTAT	low complexity	17	9
ATDTGATATDTGATDT	low complexity	16	12
SPSYSPTSPSYSPTS	low complexity	15	13
ATDTGATATDTATD	low complexity	14	12
EPVTSEPVTSEPVT	low complexity	14	10
PGPAPSPGPGPAPS	low complexity	14	10
SDSDSDSDSDSDS	low complexity	13	32
DSDSDSDSDSDSD	low complexity	13	29
PSSTEAPSSTEAP	low complexity	13	14
GSNTATQTGGNGN	low complexity	13	9
TKTVTGPTAGT	low complexity	11	13
ASASASASASA	low complexity	11	9

**Table S7.** All words of [*k* ≥ 10 AND *n* ≥ 12] in *B. rerio* (proteins <40% identical), sorted by occurrence.

**Word**	**Protein family**	***k***	***n***
DDDDDDDDDDDDDDDDDD	low complexity	18	184
QQQQQQQQQQQQQQQQQQ	low complexity	18	38
YQCKCEGLFVWPNDTCHA	EGF domain	18	22
GSFNCSCLSAFTVTDRNQ	EGF domain	18	19
AQAQAQAQAQAQAQAQAQ	low complexity	18	16
KKKKKKKKKKKKKKKKKK	low complexity	18	16
NGTEYECKCEVDHVWPSN	EGF domain	18	14
CGLNGTEYECKCEVDHVW	EGF domain	18	14
CGPNSICNNTIGSYNCSC	EGF domain	18	14
MSDPEPCRIKQEETEELI	zinc finger	18	13
YSNCTNEIGSYNCSCLDG	EGF domain	18	12
CDVITNGSCTCINGLPA	EGF domain	17	22
NGSCTCINGLPADGQFC	EGF domain	17	21
VCSLNETRYQCKCEGLF	EGF domain	17	19
ECLFSPPVCGPYSNCTN	EGF domain	17	17
THTHTHTHTHTHTHTHT	low complexity	17	17
CRELDCGAPVQVLRAA	SCRC (PF00530)	16	19
DINECEDAASVCGQYS	EGF domain	16	17
TDRNQPVSNSNPCNVC	EGF domain	16	17
TCGCIQALPSEGSLCQ	EGF domain	16	15
CDAAFDQQDAEVVCR	SCRC (PF00530)	15	25
NSIGSFNCSCLSAFT	EGF domain	15	19
IGGYMCSCWNGFNVS	EGF domain	15	15
QVCDSIVGSTCGCIQ	EGF domain	15	14
SINNTCEDVNECLKS	EGF domain	15	12
SNSNPCNVCSLNET	EGF domain	14	18
PERPPVSAPAPERP	low complexity	14	16
LTETQVKIWFQNRR	homeobox	14	12
DIDECLFSPPVCG	EGF domain	13	12
PVCGPYSNCTNE	EGF domain	12	15
PGGVGGVPGGVG	low complexity	12	13
NLPINSNNTCTD	EGF domain	12	13
LRAAAFDKGD	SCRC (PF00530)	10	13

## Figures and Tables

**Table 1.** Commonest 10 words in *Alice in Wonderland*, sorted by their occurrence, *n.*

**Word**	*n*
THE	1631
AND	865
TO	728
A	628
SHE	541
IT	530
OF	512
SAID	462
I	410
ALICE	386

**Table 2.** 10 commonest raw strings of *k* = 5 to 20 in unspaced *Alice in Wonderland*. True discrete words or phrases (DWoPs) are shaded.

**Word**	*n*
ALICE	397
SAIDT	266
AIDTH	224
SAIDTH	222
IDTHE	221
SAIDTHE	212
AIDTHE	212
ANDTH	169
THING	169
DALICE	162

**Table 3.** Top 10 contrast words of *k* = 7 to 20 in uns-paced *Alice in Wonderland*, sorted by *std(s)*, their z-score. True DWoPs are shaded. *k*: length of contrast word, *n*: occurrence, *n-L*: occurrence of left substring, *n-R*: occurrence of right substring, *n-M*: occurrence of middle substring, *std(s)*: z-score (see Introduction).

**Word**	***k***	***n***	***n*-*L***	***n*-*R***	***n*-*M***	***std*(*s*)**
ROUGHTH	7	11	14	13	114	7.44
TOTHINK	7	7	7	7	43	5.49
TOFTHEW	7	10	14	25	156	5.18
OINTHED	7	9	21	10	109	5.10
AIDNOTH	7	6	6	6	34	4.80
POFTHEE	7	5	7	6	156	4.73
DIDTHEY	7	5	9	8	221	4.67
THECOUR	7	16	16	18	52	4.45
ESAIDTO	7	26	40	77	266	4.24
RLITTLET	8	7	15	14	128	4.18

**Table 4.** Top 10 hits for ES of *k* = 5 to 20, sorted by occurrence. True DWoPs are shaded. *k*: length of raw string, *n*: occurrence, *n-L*: occurrence of left substring, *n-R*: occurrence of right substring, *n-M*: occurrence of middle substring.

**Word**	***k***	***n***	***n-R***	***n-L***	***n-M***
ALICE	5	397	397	397	401
LITTLE	6	128	128	128	128
LITTL	5	128	128	128	193
SAIDALICE	9	116	116	116	116
SAIDALIC	8	116	116	116	116
THOUGH	6	91	91	91	91
HERSELF	7	83	83	83	83
THEQUE	6	77	77	77	78
THEKING	7	62	62	62	62
HEKING	6	62	62	62	64

**Table 5.** The top 10 hits with RS-ESM of *k* = 5 to 18, sorted by their occurrence, *n*. True DWoPs are shaded. *k*: length of raw string, *n*: occurrence.

**Word**	***k***	***n***
ALICE	5	397
OFTHE	5	156
LITTLE	6	128
SAIDALICE	9	116
LIKE	4	97
THOUGH	6	91
HERSELF	7	83
THEQUE	6	77
THEKING	7	62
TURTLE	6	61

**Table 6.** Top 10 hits using CW-ESM of *k* = 5 to 18, sorted by *std(s)*, their z-score. True DWoPs are shaded. *k*: length, *n*: occurrence of the contrast word in which they are embedded, *std(s)*: z-score.

**Word**	***k***	***n***	***std***(***s***)
OFTHE	5	68	5.18
LITTLE	6	44	4.18
ALICE	5	198	3.21
SHOULD	6	14	3.20
THEMARCHHARE	12	4	3.17
THEDORMOUSE	11	9	3.09
BEGIN	5	11	2.63
WHICH	5	8	2.51
MINUTE	6	21	2.50
VENTURE	7	10	2.50

**Table 7.** Comparison of the methods described above. *TP*: True positive DWoPs detected. *Sen2*: accuracy (see Methods).

**Method**	**Hits**	***TP***	***Sen2***
*RS-ESM*, *k* = 2–18, *n* ≥ 2	1312	895	0.682
*CW-ESM*, *k* = 4–18, *n* ≥ 2	673	388	0.577
*ES*, *k* = 5–20, *n* ≥ 20	241	61	0.253
*RS*, *k* = 3–20, *n* ≥ 10	2293	540	0.235
*RS*, *k* = 5–20, *n* ≥ 20	1213	206	0.170
*CW*, *k* = 7–20, *n* ≥ 4	1927	117	0.061

**Table 8.** Words of length *k* = 12–18 in NRL3D_63, using RS-ESM. The protein family is derived from the NRL3D annotation.

**Word**	**Length**	**Protein family**	**No. of proteins**
TCNVAHPASSTKVDKKI	17	immunoglobulin	3
LLQLTVWGIKQLQAR	15	gp41	3
DATDRCCFVHDCCY	14	phospholipase	5
EKPYKCPECGKSFS	14	zinc finger domain	1 (internal repeat)
LGGTCVNVGCVPKK	14	2 kinds of reductase	3
LGRSGYTVHVQCNA	14	viral coat protein	3
TLGNSTITTQEAAN	14	viral coat protein	3
AFLGIPFAEPPVG	13	lipase/acetylcholinesterase	3
LGNGGLGRLAACF	13	phosphorylase	3
LLRISLLLIQSWL	13	growth hormone	3
TTPPSVYPLAPGS	13	immunoglobulin	3
AVLPGDGIGPEV	12	dehydrogenase	3
CLNVGCIPSKAL	12	dehydrogenase	3
FDTGSSNLWVPS	12	pepsin	3
HVQCNASKFHQG	12	viral coat protein	3
LRKAMKGLGTDE	12	annexin	3
PKDATDRCCFVH	12	phospholipase	4
QSQIVSFYFKLF	12	interferon	3
SDGIMVARGDLG	12	pyruvate kinase	3
SHVSTGGGASLE	12	phosphoglycerate kinase	3
SNASCTTNCLAP	12	phosphatase	4

**Table 9.** Summary of RS-ESM results on human texts and phylogenetic kingdoms. “euk. (eub. range)” refers to the eukaryotic proteomes that are within the same size range (in kilo-residues) as the eubacterial proteomes. Likewise, “euk. (arc. range)” refers to those within the same size range as the archaeal proteomes. For the human texts, “number of species” refers to number of texts, and average proteome size to average text size (in kilo-characters).

**Superkingdom**	**Number of species**	**Total proteins**	**av. proteome size (kres)**	**av. protein len. (res)**	**Words/kres**	**Words/prot**
eukarya	36	382698	4902	461	1.615	0.745
euk. (eub. range)	7	15205	1025	472	0.957	0.452
euk. (arc. range)	4	3459	374	432	0.424	0.183
eubacteria	35	104006	947	319	0.670	0.214
archaea	28	65197	681	292	0.665	0.194
human texts	9	N/A	1322	N/A	13.939	N/A
